# Moving towards elimination: findings from the South Africa prevention of mother to child transmission evaluation (SAPMTCTE)

**DOI:** 10.1186/s12879-019-4334-3

**Published:** 2019-09-16

**Authors:** Louise Kuhn, Ameena E. Goga

**Affiliations:** 1 Gertrude H. Sergievsky Center, College of Physicians and Surgeons, Columbia University Medical Center, 630 W 168th Street, New York, NY 10032 USA; 2 Department of Epidemiology, Mailman School of Public Health, Columbia University Medical Center, 630 W 168th Street, New York, NY 10032 USA; 3 Health Systems Research Unit, South African Medical Research Council, Pretoria, 0001 South Africa; 4 Department of Paediatrics, University of Pretoria, Pretoria, 0001 South Africa

It is remarkable that we find ourselves at a point in history where we can seriously consider the possibility of virtual elimination of mother-to-child HIV transmission (MTCT). Effective antiretroviral strategies, carefully honed through randomized clinical trials, provide the means to almost entirely prevent the transmission of HIV from an HIV-infected woman to her infant during pregnancy, delivery and breastfeeding - if started timeously and sustained through the full period of risk [1]. Massive mobilization of governments, policy-makers, health service managers, clinicians, researchers and communities have led to implementation of Prevention of Mother To Child Transmission (PMTCT) interventions on an impressive scale [1]. South Africa, which faces the world’s highest adult HIV prevalence, has risen to the challenge, also implementing the one of the world’s largest antiretroviral drug treatment program [2]. In so doing, South Africa has made impressive gains in mitigating the tragedy of its generalized and substantial HIV epidemic.

Program evaluation and its component parts, now often subsumed in the rubric of “implementation science,” is a critical piece of this success. Implementation science in this context has two main objectives: (1) quantifying success (or failure) along the path towards achieving elimination of MTCT; and (2) identifying gaps and weaknesses in the health system that can be improved to achieve the desired outcomes. This Special Issue is devoted to describing methods and findings of the South Africa Prevention of Mother to Child Transmission Evaluation (SAPMTCTE). This landmark study, conducted over a more than four year period, was led by the South African Medical Research Council in collaboration with the South African National Department of Health, the US Centers for Disease Control, the United Nations Children’s Education Fund (UNICEF) and other South African university partners.

SAPMTCTE provided the first nationally-representative data in a high-burden country demonstrating dramatic reductions in vertical HIV transmission achieved as a result of implementation of PMTCT programs in South Africa [3, 4]. The national estimate of early HIV transmission (detectable by 4–8 weeks) was 3.5% in 2010, further improving to 2.6% by the 2012–13 round of the survey [3, 4]. HIV infection detectable by 4–8 weeks reflects transmission that occurred intrauterine, intrapartum and through the first weeks of breastfeeding. Had antiretroviral drug interventions not been in place, we know that HIV transmission by this timepoint is likely to have occurred in almost a quarter of infants born to HIV-infected mothers [5].

In the first paper in this special issue, Goga et al. (Paper 1) address a fundamental question: “What will it take for the Global Plan priority countries in Sub-Saharan Africa to eliminate mother-to-child transmission of HIV?” To tackle this question, the authors compare characteristics of four countries (Thailand, Cuba, Belarus and Armenia) that have met the World Health Organization (WHO) criteria for elimination of MTCT, to 22 countries prioritized by the Global Plan for MTCT elimination, including South Africa and all of its neighbors, who have not yet achieved the elimination criteria. The authors apply three frameworks for considering how improved health outcomes can be attained in a population. These include WHO’s building blocks to strengthen health systems [6], van Olmen’s Health System Dynamics framework [7] and Baral’s socio-ecological model for HIV risk [8]. Importantly, research and information are central to each of these frameworks.

In the next paper, Jackson et al. (Paper 2) provide a detailed presentation of the over-arching methodological approach of the three national studies. SAPMTCTE builds on a fine tradition in implementation science related to PMTCT that optimally utilizes laboratory testing of dried blood spots to determine health system related outcomes. Specifically, using the approach first introduced by Rollins et al., a first round HIV antibody screen of *infants’* dried blood spots identifies the infants’ *mothers’* HIV status and then among HIV-exposed infants only, virological testing using HIV diagnostic PCR determines the *infants’* HIV status (transmission rate) [9]. This simple approach demonstrated feasibility and acceptability at national level, facilitating implementation science research on monitoring PMTCT effectiveness and impact.

But SAPMTCT takes several steps further to strengthening inferences including paying careful attention to population sampling. Jackson et al. (Paper 2) describe the practical application of well thought-out statistical approaches for attaining population-representative estimates and the appropriate weighting methods for use in the analysis. Importantly, regional (provincial) estimates of the transmission rate are possible. This is a great advance for interpreting the findings and strengthening our confidence in the primary findings which are one of remarkable success in attaining such low rates of early transmission, fairly consistently across almost all of the Provinces even those with some of the fewest resources [3, 4].

Ngandu et al. (Paper 3) report on the Infrastructural and human-resource factors associated with return of infant HIV test results to caregivers. Such analyses are important as appropriate postnatal follow up and early initiation of HIV positive children on triple antiretroviral therapy is contingent on timely return of the HIV test results to caregivers. This paper illustrates that returning infant HIV test results to caregivers within 3 weeks of blood draw was only reported in 26% of facilities. The analyses underpin the need for quicker return of results to primary health care facilities, and the authors hypothesise that better liaison between laboratories and facilities are needed to optimize quick return of results to caregivers.

Singh et al. (Paper 4) delight us with a discussion of the practical application of mobile technology in the data collection and quality control in SAPMTCTE. Cheap cell phones, not “smart” enough to be considered worth stealing, were “smart” enough to be tremendously efficient and accurate in collecting the data for the study. Between 2010 and 2013, data from 10,554, 10,071, and 10,536 interviews, each with about 186 variables, were successfully uploaded from 151 cell phones, collecting data at 580 health facilities in 51 districts, across all nine provinces of South Africa. Error rates and failure rates were exceptionally low challenging skeptics still hankering after the good ol’ days of paper and pencil.

Drug resistance is an interesting marker as it tracks both the success and the failure of PMTCT. In the dwindling numbers of new infant HIV infections, Hunt et al. (Paper 5) measure the frequency of viral mutations which confer resistance to the antiretroviral drugs currently used as part of first-line adult treatment (efavirenz) and as part of infant prophylaxis (nevirapine), at six weeks postpartum, and report that they are common. This is tells us that uptake of antiretroviral drug treatment and prevention programs is high (success) resulting in very few new infections (success) albeit one where viral resistance mutations are the norm. First-line treatment recommendations for infants favor utilizing the boosted protease-inhibitor ritonavir/lopinavir [10, 11]. Ritonavir/lopinavir-based regimens were tested initially because of fears of drug resistance, but were found to have better outcomes even in the absence of prior drug exposure [10, 11]. Reassuringly, Hunt et al. observe that the profile of resistance mutations in the new era of maternal antiretroviral treatment and longer infant prophylaxis regimens is no worse than that observed in the single-dose nevirapine era [12].

SAPMTCTE also added on a longitudinal, observational epidemiological study to track post-natal transmission and survival to 18 months. This component draws our attention to one of the weakest links in the PMTCT cascade – postnatal follow-up and care.

Ngandu et al. (Paper 6) quantify attrition from the study – which was high at 18 months (31.0%). This, sadly, is likely to reflect attrition from the program. While we might hope that those no longer part of the study are representative of those who remain, this is unlikely to be the case. As Ngandu et al. demonstrate higher frequency of missed visits occur amongst mothers not on triple antiretroviral therapy (ART). As a result, national estimates of 18 month HIV-free survival rates are open to biases that cannot be analyzed away. PMTCT and child health programmes will need to give more thought to the methodology of how to ascertain this critical endpoint in an unbiased way.

Larson et al. (Paper 7) measure reported rates of maternal adherence to antiretroviral drugs postnatally and maternal report of infant adherence to postnatal prophylaxis in the retained cohort. These rates of adherence are subject to similar biases that affect overall attrition and, as a result, adherence is probably worse than it seems. This is particularly concerning in the current context where lifelong ART is recommended for all pregnant and lactating women, with 6 weeks of infant prophylaxis and breastfeeding to age 24 months.

Both Ngandu et al. and Larson et al. (Papers 6 and 7) identify younger maternal age as a risk factor for attrition and poor adherence. The combined social and biological vulnerability of adolescent girls and young women in terms of new acquisition of HIV infection has rightfully received a great deal of attention in South Africa [13]. The work here highlights yet further vulnerability of the young women already infected with respect to engagement and retention in the health care system – gaps that will adversely affect their own and their children’s health. A previous analysis of SAPMTCTE also identified new primary infections in young pregnant women and in young women soon after delivery as key drivers of HIV transmission in infants [14]. Surely, we should heed this as a call to action – on the path to eliminating vertical transmission in infants, the needs of young women, both those already infected and those at risk of infection, urgently need attention.

Once full elimination of HIV transmission is achieved, the sobering case series describing the poor uptake of antiretroviral drugs in HIV infected infants by Mathivha et al. (Paper 8) will remain a matter of the past. As coverage of PMTCT programs increase, infants are less and less likely to acquire infection and those who do have more and more overlapping biological and social risk factors. Weak health systems, exemplified in the long turn-around times of laboratory results described by Ngandu et al. (Paper 3) add to these problems. Although few in number, these new infant infections reflect a high burden of suffering, and delayed access to treatment amongst children located in varying geographical settings, nationally. But since few in number, health planners need to consider pathways to ensure that these high risk infants can access specialist care (that does exist in all urban centers in South Africa) earlier, with benefits of more intensive and informative laboratory monitoring, and potential to be treated, if appropriate, with recently-approved, more expensive antiretroviral drugs [15].

This collection of papers describing methods and findings from SAPMTCTE are a welcome reminder of how far we have come. The findings are also a reminder of how far we have to go to eliminate MTCT in South Africa, and, in the interim, how far we have to go to ensure timely, early ART initiation among children who acquire HIV infection. We are informed that given the high prevalence of HIV in pregnant women in South Africa - which is currently estimated at just under a third – even if low enough transmission rates at 18 months can be attained, the numbers of new infections per 100,000 live births (regardless of HIV status) may exceed the targets set by WHO. The large denominator of HIV-infected women of child-bearing age also reminds us of the considerable burden of sustaining such a large PMTCT program essentially indefinitely. Continued and repeated high quality evaluations will be essential. Most importantly, the most vulnerable need attention. This includes young women who continue to be at high risk of HIV acquisition and lack of engagement and retention in care, and the rare infants who acquire infection despite the heroic efforts. In addition to the current metrics to assess attainment of milestones to achieve virtual elimination of MTCT, one wonders whether equity ought to be included. Disparities across and within regions, and across subgroups of the population highlight inequities within the society. Addressing these inequities is likely to have synergistic benefits for all in the long run.
